# Hyperintense lesions of the middle cerebellar peduncle and beyond: a pictorial essay

**DOI:** 10.1590/0100-3984.2024.0001

**Published:** 2025-01-10

**Authors:** João Vitor Gerdulli Tamanini, Guilherme Antonio Silva Ribeiro, Adriana Tami Kimura, Luiz Fernando Borella, Tomás de Andrade Freddi, Fabiano Reis

**Affiliations:** 1 Escola Paulista de Medicina da Universidade Federal de São Paulo (EPM-Unifesp), São Paulo, SP, Brazil; 2 Faculdade de Ciências Médicas da Universidade Estadual de Campinas (FCM-Unicamp), Campinas, SP, Brazil; 3 Hospital do Coração (Hcor), São Paulo, SP, Brazil

**Keywords:** Middle cerebellar peduncle, Multiple sclerosis, Neuromyelitis optica, Encephalomyelitis, acute disseminated, Myelinolysis, central pontine, Pedúnculo cerebelar médio, Esclerose múltipla, Neuromielite óptica, Encefalomielite aguda disseminada, Mielinólise central da ponte

## Abstract

The middle cerebellar peduncle (MCP) is the largest afferent system of the cerebellum and consists of fibres from the cortico-ponto-cerebellar tract. Specifically, several relevant diseases can present with hyperintensity in the MCP on T2-weighted/fluid-attenuated inversion recovery (T2/FLAIR) magnetic resonance imaging sequences, including multiple sclerosis; acute disseminated encephalomyelitis; neuromyelitis optica spectrum disorder; progressive multifocal leucoencephalopathy; hepatic encephalopathy; osmotic demyelination syndrome; multiple system atrophy; fragile X-associated tremor/ataxia syndrome; megalencephalic leucoencephalopathy with subcortical cysts; spinocerebellar ataxias; hemi-pontine infarct with trans-axonal degeneration; and diffuse midline glioma with the histone H3K27M mutation. The aim of this pictorial review is to discuss the imaging findings that are relevant for the differential diagnosis of diseases presenting with MCP hyperintensity on T2/FLAIR sequences. Such knowledge is of utmost importance for the practicing radiologist.

## Abstract (pt)

O pedúnculo cerebelar médio (PCM) é o maior sistema aferente do cerebelo e consiste de fibras do trato corticopontocerebelar. Especificamente, diversas doenças relevantes podem se apresentar com hipersinal em T2/FLAIR no PCM. Dentre elas, é possível citar esclerose múltipla, encefalomielite aguda disseminada, doenças do espectro da neuromielite óptica, leucoencefalopatia multifocal progressiva, encefalopatia hepática, síndrome da desmielinização osmótica, atrofia de múltiplos sistemas, síndrome de tremor e ataxia associada ao X frágil, leucoencefalopatia megalencefálica com cistos subcorticais, ataxias espinocerebelares, infarto hemipontino com degeneração transaxonal e glioma difuso de linha média com mutação de histona H3K27M. O objetivo dessa revisão iconográfica é discutir os achados de imagem dos principais diagnósticos diferenciais de doenças que se apresentam com hipersinal em T2/FLAIR no PCM, um conhecimento de grande relevância para o radiologista.

## INTRODUCTION

**Figure 1:**
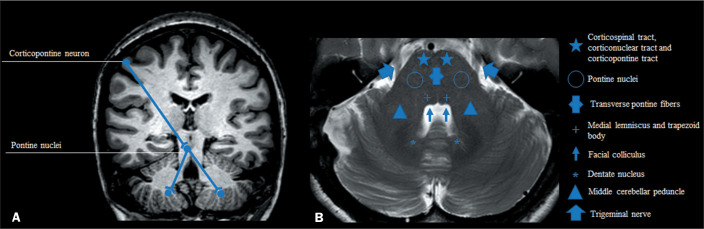
Coronal and axial views (A and B, respectively) showing the cortico-ponto-cerebellar pathways.

The middle cerebellar peduncle (MCP) is the largest afferent system of the cerebellum and consists of fibres from the cortico-ponto-cerebellar tract (Figure 1). Lesions in this structure can be detected by brain magnetic resonance imaging (MRI) as hyperintensity on T2-weighted/fluid-attenuated inversion recovery (T2/FLAIR) sequences.

Several aetiologies may present with FLAIR hyperintensity in the MCP: demyelinating diseases-multiple sclerosis (MS), acute disseminated encephalomyelitis (ADEM), neuromyelitis optica spectrum disorder (NMOSD) and progressive multifocal leucoencephalopathy (PML); toxic-metabolic diseases-hepatic encephalopathy (HE) and osmotic demyelination syndrome (ODS); a degenerative disease-multiple system atrophy (MSA); genetic diseases -fragile X-associated tremor/ataxia syndrome (FXTAS), megalencephalic leucoencephalopathy with subcortical cysts (MLC) and spinocerebellar ataxias (SCAs); a vascular disease-hemi-pontine infarct with trans-axonal degeneration; and a neoplastic disease-diffuse midline H3K27M-mutated glioma.

The purpose of this pictorial review is to discuss the imaging findings that are relevant for the differential diagnosis of diseases presenting with T2/FLAIR hyperintensity in the MCP on T2-weighted imaging (T2WI). Such knowledge is of utmost importance for the practicing radiologist.

## DISEASES OF ADULTHOOD

### Demyelinating diseases

#### MS

A chronic autoimmune disease of the central nervous system (CNS), MS presents with inflammation, demyelination and axonal loss. The female-to-male ratio in MS is approximately 3:1, and the average age at onset is 20-40 years^(1)^.

**Figure 2:**
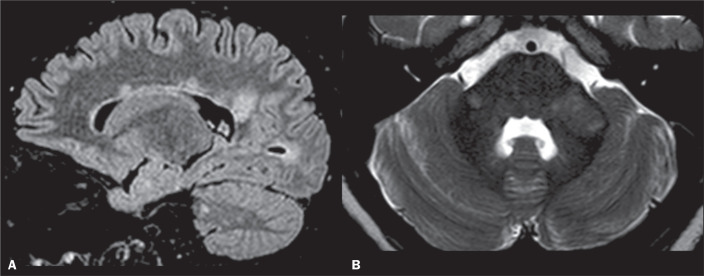
MS. A: Sagittal FLAIR sequence showing callosal lesions perpendicular to the ventricular wall. Note also the lesions in the occipital and cerebellar white matter. B: Axial T2WI showing bilateral hyperintense lesions (more conspicuous on the left side) involving the MCPs.

With regard to MRI findings, the lesions are usually isointense to hypointense on T1-weighted imaging (T1WI) and hyperintense on T2/FLAIR sequences (Figure 2). The McDonald criteria for MS include dissemination in time and space that can be identified with MRI. Specifically, dissemination in space requires one or more lesions that are hyperintense on T2WI in two or more of the following locations: periventricular; cortical or juxtacortical; infratentorial; or in the spinal cord. Dissemination over time requires either new lesions that are hyperintense on T2WI when compared with a previous MRI or the simultaneous presence of gadolinium-enhancing and non-enhancing lesions^(2)^. A classic T2/FLAIR finding consists of lesions oriented perpendicular to the lateral ventricles and that are well-depicted on parasagittal images (Figure 2A). These alterations are referred to as Dawson’s fingers. Active MS lesions show contrast enhancement and usually form an incomplete pattern around the periphery to create an open ring sign. Such lesions are frequently infratentorial, periventricular or juxtacortical (Figure 2). Mixed white and grey matter lesions can also be identified. The MCP is an important tract of white matter and is one of the most commonly affected structures in MS^(1,3)^, as depicted in Figure 2B and detailed in Table 1.

**Table 1:** Epidemiology and imaging characteristics of the various diseases affecting the MCP.

Group	Disease	Usual age at presentation (years)	Most commonly affected gender	Distinguishing characteristics in imaging
	MS	20-40	Female	T2/FLAIR hyperintense lesions oriented perpendicular to the lateral ventricles
	ADEM	5-8	Male	Bilateral, asymmetric, poorly marginated, multifocal lesions, presenting with T2 hyperintensity
Demyelinating diseases	NMOSD	Approximately 40	Female	Characteristically involving the optic chiasm and retrochiasmatic regions, with lesions typically found in aquaporin 4-rich areas such as the periependymal regions abutting the ventricles
	PML	Depends on the specific cause leading to immunosuppression; most commonly seen in patients with AIDS, with CD4 counts of 50-100 cells/pL; potentially related to immunosuppressive monoclonal antibody therapy		Discrete multifocal, asymmetric, periventricular and subcortical involvement, with no significant mass effect or contrast enhancement
Toxic metabolic diseases	HE	Depends on the aetiology of liver dysfunction and portal hypertension		T2/FLAIR symmetric hyperintensity in the insula, thalamus and posterior limb of the internal capsule; hyperintensity on T1WI in the globus pallidus and anterior midbrain
	ODS	Depends on the aetiology of the osmotic stress affecting oligodendroglial cells		Restricted diffusion in the pons; hyperintense signal on T2/FLAIR in the central portion of the pons, sparing the periphery
Degenerative diseases	MSA	54-61	Male	Hot cross bun sign in the pons on T2; disproportionate atrophy in the olivary nuclei and MCP
	FXTAS	Approximately 60 for tremor and ataxia	Male	T2/FLAIR hyperintense lesions in the MCPs and in the splenium of the corpus callosum
Genetic diseases	MLC	5	Male	Subcortical cysts, permanence of the cavum septum pellucidum, symmetric signal alteration of white matter
	SCA3	5-70 (median, 40)	Male	Pontine and cerebellar atrophy; atrophy of the globus pallidus atrophy in some patients with long-standing disease
Vascular diseases	PI with Wallerian degeneration	Depends on the stroke aetiology		Paramedian pontine hyperintensity on T2WI
Neoplastic diseases	Diffuse midline H3K27M-mutated glioma	5-11	No difference	T2 hyperintense lesion in the pons, thalamus or spinal cord

#### NMOSD

One CNS autoimmune disease that causes multifocal inflammation, mostly in the optic nerves and spinal cord, is NMOSD. The median age at onset is 40 years, with 20% of patients being children or adults > 65 years old. Most anti-aquaporin-4-positive patients are female, whereas the male-to-female ratio is closer to 1:1 in those who are seronegative. Clinically, NMOSD has six core presentations-area postrema syndrome, acute brainstem syndrome, diencephalic syndrome, symptomatic cerebral syndrome, optic neuritis and myelitis-all of which can be seen during acute attacks and relapses^(4)^.

**Figure 3:**
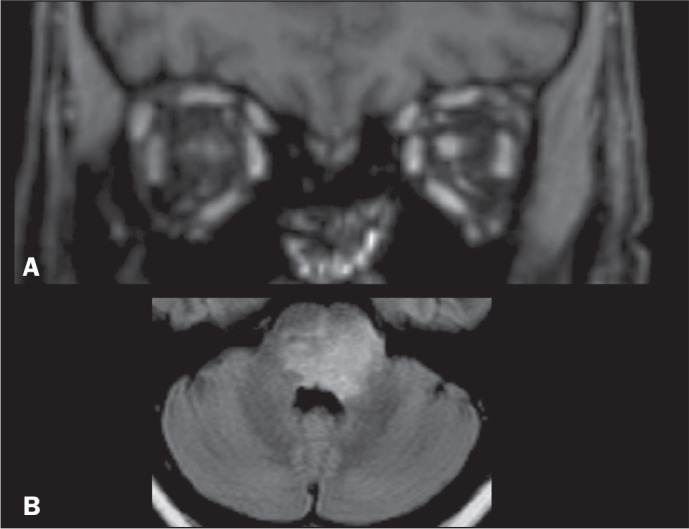
NMOSD. A: Contrast-enhanced coronal T1WI of a patient with symptoms of acute optic neuritis of the left eye showing a contrast-enhanced lesion in the intraorbital portion of the left optic nerve. B: Axial FLAIR sequence showing a hyperintense confluent lesion in the pons and in the MCPs. Note the involvement of the periventricular regions adjacent to the fourth ventricle in aquaporin-4-rich areas of the periependymal tissue.

When NMOSD is suspected, the imaging modality of choice is MRI. Specifically in acute phases, orbit MRI can show hyperintense lesions in the optic nerves, with chiasmatic and retrochiasmatic involvement (Figure 3A). A T2WI scan of the brain often reveals hyperintense lesions around the ventricles and in the MCP (Figure 3B), areas that are rich in aquaporin-4. In addition, MRI of the spinal cord typically shows longitudinally extensive myelitis involving three or more contiguous medullary segments. There can be associated cord swelling, and central grey matter involvement is usual. After contrast administration, T1WI of the spinal cord can show patchy cloud-like enhancement of lesions that appeared as bright spots on T2WI. There can be involvement of the MCP^(3,4)^, as illustrated in Figure 3B and described in Table 1.

#### PML

The CNS demyelinating disease known as PML is caused by the John Cunningham virus. This virus is typically dormant in healthy individuals but can be reactivated by impaired cellular immunity, as seen in patients infected with HIV or who are using drugs such as natalizumab. Usually progressing over the span of a few months, PML results in neurological impairment and dementia. Common symptoms include paresis, language disorders and ataxia^(5,6)^.

**Figure 4:**
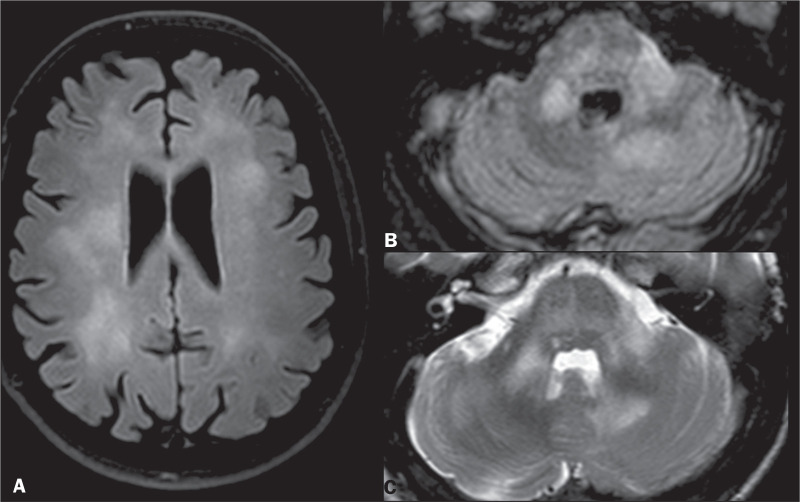
PML. A: Axial FLAIR sequence showing periventricular and subcortical white matter lesions, with no mass effect. T1WI after contrast (not shown) revealed no enhancement. Axial FLAIR sequence (B) and T2WI (C) showing pontine and cerebellar white matter lesions, as well as hyperintensities in both MCPs.

The MRI lesions can be seen as T2/FLAIR hyperintense multifocal, bilateral, and asymmetric alterations (Figure 4) that are usually located in the cerebral white matter (most often subcortically), cerebellum and brainstem. Crescent-shaped cerebellar lesions are characteristic findings. The lesions can also affect the basal ganglia or thalamus but do not usually present oedema, nor do they present a mass effect or enhance with contrast. This pattern of T2 hyperintensity, with no mass effect or contrast enhancement and restricted patchy peripheral diffusion, is also observed in the MCP^(6)^, as indicated in Figure 4B, Figure 4C, and Table 1.

### Toxic metabolic diseases

#### HE

Acute or chronic liver failure can cause the brain dysfunction known as HE^(7)^, which frequently presents as personality alterations, as well as changes in cognition, consciousness and motor function. The pathophysiology of this condition is complex and involves blood-derived factors that alter the normal working of the brain and blood-brain barrier^(8)^.

**Figure 5:**
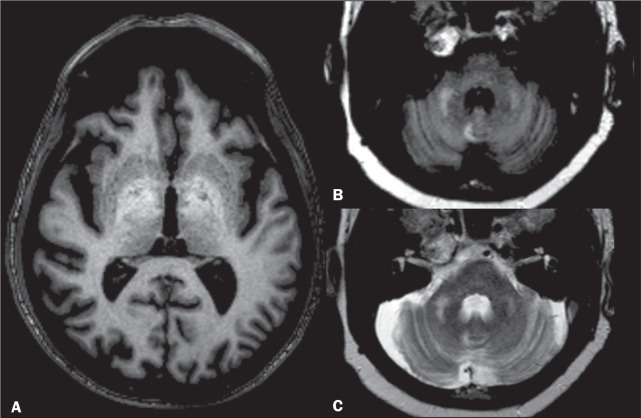
HE. A: Axial T1WI showing hyperintensities in the globus pallidus. Axial FLAIR sequence (B) and axial T2WI (C) showing asymmetrical hyperintensities in the MCPs.

Neuroimaging with T2/FLAIR shows symmetric hyperintensity in the insula, thalamus, cingulate gyrus and posterior limbs of the internal capsule. In more severe cases, there is diffuse cortical oedema and T2/FLAIR hyperintensity. Diffusion-weighted imaging (DWI) can show alterations and distribution similar to those found on T2/FLAIR. On T1WI, hyperintensity is commonly observed in the globi pallidi because of manganese deposition (Figure 5A). The MCP can show bilateral symmetrical involvement (Figure 5B,C; Table 1).

#### ODS

The metabolic disease known as ODS consists of cell shrinkage and demyelination in response to osmotic stress. Brain areas that are rich in oligodendrocytes and myelin tend to be the most affected. It frequently follows rapid correction of hyponatremia but can also be related to hypokalemia, hypophosphatemia, malnutrition, harmful alcohol use and liver dysfunction. Clinical features include impaired vigilance, dysphagia, dysarthria and limb weakness^(9)^.

**Figure 6:**
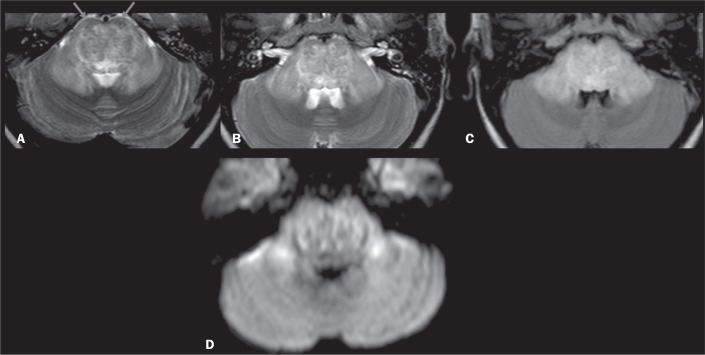
ODS. Axial T2WI (A,B) and axial FLAIR sequence (C), showing confluent hyperintensity involving the pons while sparing its anterior peripheral aspect and extending into the MCPs. D: DWI showing restricted diffusion

The diagnostic modality of choice is brain MRI, with the areas of demyelination showing hyperintensity on T2/FLAIR and hypointensity on T1 (Figure 6). Characteristically, ODS involves the central pons and spares the corticospinal tract, peripheral pons and tegmentum, thereby creating a trident pattern signal in the anterior pons. In some acute cases, there is restricted diffusion (Figure 6D), although this differs from ischemic lesions that extend to the periphery of the pons while sparing the midline. The lesions can extend to the MCP^(10)^ (Figure 6; Table 1).

### Degenerative diseases

#### MSA

The neurodegenerative disorder MSA is an α-synucle-inopathy that presents with a combination of parkinsonism, cerebellar ataxia, autonomic nervous system dysfunction and cognitive deficits, as well as pyramidal and extra-pyramidal signs. The mean age of onset is 54-61 years. It can be divided into two clinical subtypes^(11)^: MSA with parkinsonism as the predominant manifestation; and MSA with cerebellar ataxia as the predominant manifestation.

**Figure 7:**
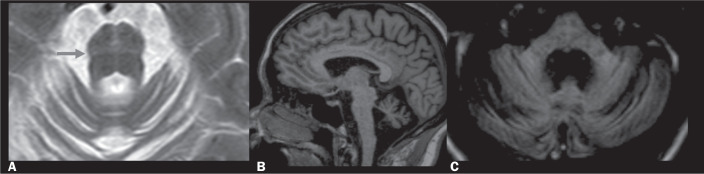
MSA with cerebellar ataxia. A: Axial T2WI showing a hyperintense cross in the form of a hot cross bun. B: Sagittal T1WI showing flattening of the anterior aspect of the pons and cerebellar atrophy. C: Axial FLAIR sequence showing cerebellar and MCP atrophy, together with hyperintensities in the MCPs.

On MRI, atrophy of the pons, cerebellum and MPC can be seen (Figure 7B). In addition, the characteristic “hot cross bun” sign, which consists of cruciform T2 hyperintensity in the pons, can be seen (Figure 7A). However, this sign is not specific for MSA as it occurs in other cerebellar degenerative disorders. Other alterations include lateral putaminal rim hyperintensity on T2WI and T2 hyperintensity in the posterior putamen in MSA with parkinsonism as the predominant manifestation^(11)^.

### Genetic diseases

#### SCAs

The group of genetic diseases known as SCAs consists of neurodegenerative diseases that frequently manifest as cerebellar ataxia, slurred speech, ocular motor abnormalities and a wide range of other neurological features^(12,13)^. The prevalence is estimated to be between 1 and 5 per 100,000 population^(12)^. They can be caused by dynamic repeat expansion mutations and non-repeat mutations^(13)^, with the most common forms being caused by repeat expansions, including SCA1, SCA2, SCA3, SCA6, SCA7 and SCA17^(13)^. The most common of those is SCA3, also known as Machado-Joseph disease^(13)^.

**Figure 8:**
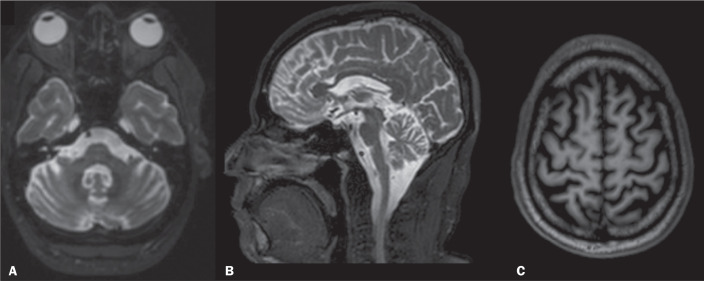
SCA3 in a 29-year-old man. Axial and sagittal T2WIs (A and B, respectively) demonstrating atrophy of the cerebellar hemispheres, as well as of the vermis, pons and MCPs. C: Axial T1WI showing atrophy of the cerebral cortex.

The MRI findings for SCA3 include atrophy of the infraand supra-tentorial structures, with brainstem and cerebellar volumetric reductions (Figure 8) being common findings^(14)^. In the cerebellum, atrophic changes in the vermis and superior peduncle are noticeable, whereas pontine atrophy is more conspicuous in the tegmentum^(15,16)^. Other sites of atrophy include the frontal and temporal lobes, the globus pallidus, the dentate nucleus and the red nucleus^(16)^. As noted in Table 1, T2 hyperintensity of the transverse pontine fibres and MCP is also observed^(16)^.

### Vascular diseases

#### Pontine infarction with Wallerian degeneration

Pontine infarction (PI) is a consequence of vascular obstruction hampering proper blood flow to the pons. Clinically, PI can present with manifestations ranging from pure motor/sensory contralateral deficits, ipsilateral cranial nerve palsy associated with contralateral motor and/or sensory impairment or even locked in syndrome^(17)^. There are no precise epidemiological studies of this condition, and PIs of different aetiologies show different epidemiological profiles; they can be caused by small vessel disease, cardiac emboli or large artery atherosclerosis^(17)^. After the ischemic insult, Wallerian degeneration occurs in axons distal to the site of neuronal lesion, leading to demyelination and disintegration^(18)^. This, in turn, leads to morphological alterations of the MCP. These demyelination events result in hyperintensity on T2/FLAIR MRI sequences of the affected structures. As previously mentioned, the MCP contains fibres of the cortico-ponto-cerebellar tracts that originate in the contralateral pontine nuclei. Unilateral pontine lesions affect the ipsilateral cortico-pontine fibres and the crossing contralateral ponto-cerebellar fibres^(19)^.

**Figure 9:**
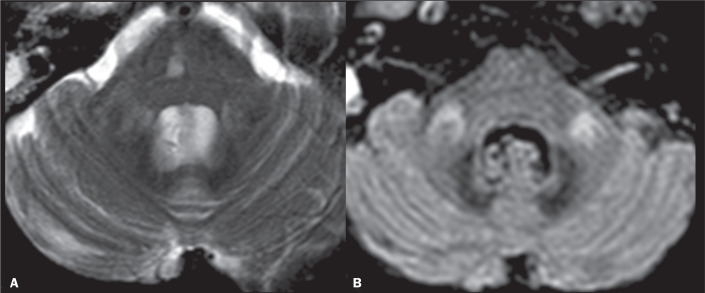
Wallerian degeneration. A: Axial T2WI showing hyperintensity (suggestive of sequelae of infarction) in the right hemi-pons. B: Axial FLAIR sequence showing hyperintensity in both MCPs. These findings are suggestive of Wallerian degeneration caused by a PI.

Imaging findings for PI and posterior Wallerian degeneration depend on the specific phase of the process. Specifically, in the first few minutes of the ischemic process, there is an increase in the intensity of the signal on DWI and a decrease in the apparent diffusion coefficient in the MCP, inferolateral portion of the pons, flocculus, and anteroinferior surface of the cerebellum. At 6 h after symptom onset, T2/FLAIR hyperintensity is detectable (Figure 9A). As it progresses, there is symmetric, bilateral T2/FLAIR hyperintensity of the MCP, albeit without gadolinium enhancement, although there can be volume loss (Figure 9B; Table 1).

## DISEASES OF CHILDHOOD

### Demyelinating diseases

#### ADEM

One immune-mediated demyelinating CNS disorder, known as ADEM, which is most commonly diagnosed in boys between 5 and 8 years of age, has been associated with previous infection^(20,21)^. It is also a common manifestation of anti-myelin oligodendrocyte glycoprotein disease.

**Figure 10:**
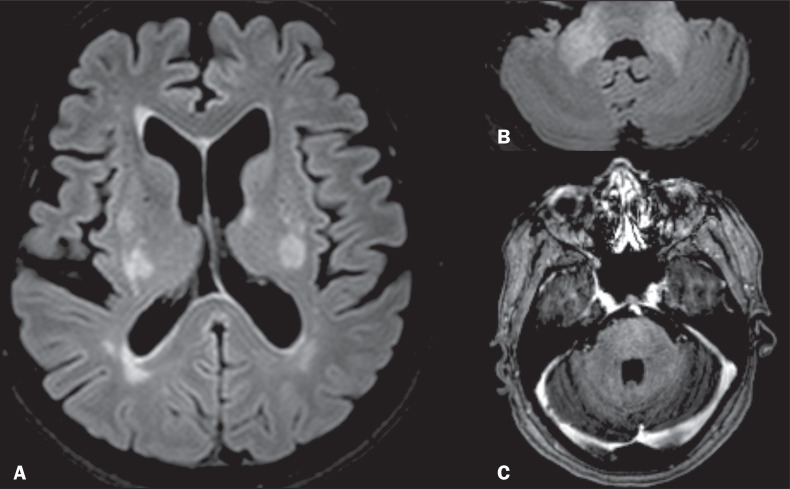
ADEM. A: Axial FLAIR sequence demonstrating supratentorial lesions involving the white and grey matter (basal ganglia and left thalamus). B: Axial FLAIR sequence depicting bilateral hyperintense MCP lesions. C: Contrast-enhanced T1WI showing no enhancement in the MCP lesions.

Neuroimaging on T2/FLAIR frequently reveals multiple, bilateral, poorly delimitated hyperintense lesions with surrounding oedema (Figure 10). These lesions are usually located in subcortical and central white matter regions, as well as in the thalami, brainstem, cerebellum, basal ganglia and grey-white matter junction (Figure 10). Typically, the brain lesions in a given case of ADEM all show a similar stage of development. In this regard, most lesions show similar degrees of enhancement in response to contrast, resulting in a ring pattern. This finding is useful in distinguishing ADEM from MS, in which focal lesions that show gadolinium enhancement coexist with lesions without enhancement^(22)^. In addition, DWI can reveal restricted diffusion at the periphery and there can be involvement of the MCP^(3)^, as illustrated in Figure 10B and described in Table 1.

### Genetic diseases

#### FXTAS

Fragile X-associated conditions include several diseases associated with pathogenic variants of the FMR1 gene, with the most common form being the fragile X syndrome caused by more than 200 CGG repeats in FMR1. Fragile X syndrome usually presents with intellectual disability, developmental delay, autism spectrum disorder and seizures^(23)^. In contrast, FXTAS is caused by 55-200 CGG repeats of the FMR1 gene and manifests as kinetic tremor, gait ataxia, executive dysfunction and neuropathy^(23)^; this condition is more common in men^(24)^.

**Figure 11:**
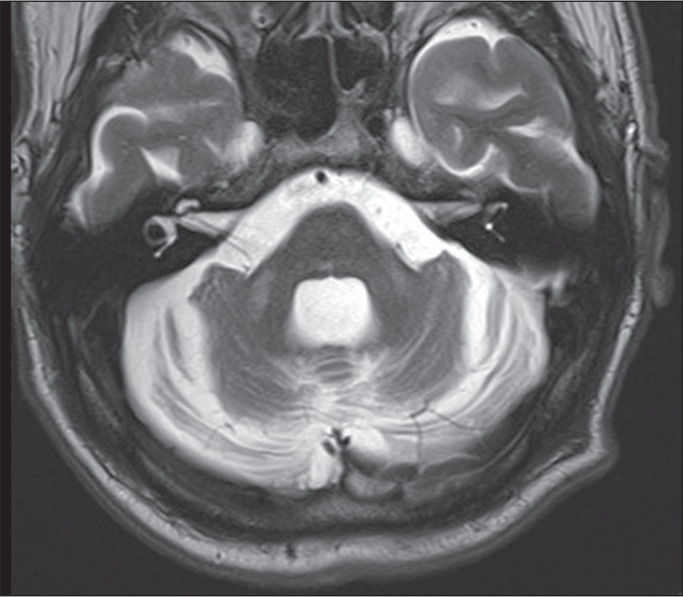
FXTAS. Axial T2WI showing MCP hyperintensity and cerebellar atrophy.

There are two major neuroimaging findings in FXTAS^(24)^: T2 hyperintensity in the MCP (Figure 11), which is seen in 60% of males and 13% of females; and T2 hyperintensity in the corpus callosum, the male-to-female ratio of which is similar to that reported for T2 hyperintensity in the MCP^(25)^. Other imaging findings in FXTAS include T2 hyperintensity in the pons, insula and periventricular region, as well as generalised brain and cerebellar atrophy (Table 1).

#### MLC

Another genetic disorder of childhood is MLC, which manifests as cerebral white matter oedema. There are two different forms of the disease. The first form, known as classic MLC, presents with a deteriorating phenotype and is mostly associated with MLC1 mutations, whereas the second form presents with a remitting phenotype and is associated with GLIALCAM gene mutations. Patients with the deteriorating form present with macrocephaly, ataxia, spasticity and epilepsy, whereas those with the remitting form present with neurological deterioration^(18)^. In either form, MLC predominantly affects males, with the initial motor deterioration being detected at a median age of 5 years^(26)^.

**Figure 12:**
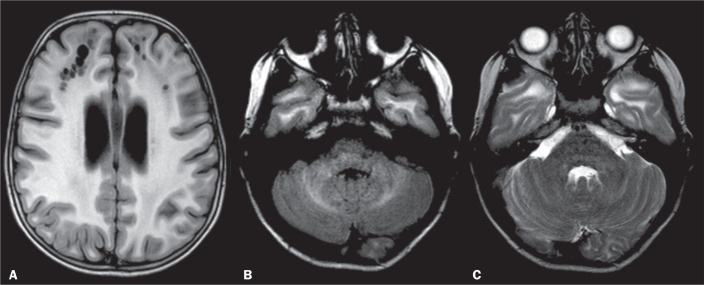
MLC. A: Axial T1WI showing cysts in the white matter frontal lobes and persistence of the cavum septum pellucidum. Axial FLAIR sequence (B) and axial T2WI (C) showing a mildly hyperintense signal in the cerebellar white matter (and in the MCPs), together with diffuse white-matter abnormalities in the temporal and occipital lobes.

In many patients with MLC, one can notice oedema and signal abnormalities in the cerebral and cerebellar white matter. With optic radiation sparing, the MCP can also be altered (Figure 12B,C). As the disease progresses, there can be enlargement of the ventricles and subarachnoid spaces^(26)^. On DWI, abnormal white matter can show increased diffusion^(27)^. In addition, subcortical cysts, with or without near-cyst rarefaction of subcortical white matter, primarily in the anterior temporal lobe and frontoparietal regions, are common (Figure 12A). There can be persistence of the cavum septum pellucidum. Reversal of the MRI abnormalities can occur in patients with the remitting form of MLC^(26)^, as described in Table 1.

### Neoplastic diseases

#### Diffuse midline H3K27M-mutated glioma

Gliomas are the most common form of primary CNS tumours^(28)^. Among them, diffuse midline H3K27M-mutated gliomas form a group of high-grade neoplastic lesions that arise from midline structures such as the thalamus, brainstem and spine^(28)^. These tumours are most commonly identified in the paediatric population, with a median age at presentation of 5-11 years^(29)^; males and females are equally affected^(29)^. Patients usually present with cerebrospinal fluid obstruction, with or without brainstem dysfunction that can include ataxia, pyramidal signs and cranial nerve abnormalities; thalamic lesions can lead to motor weakness and gait disturbance^(30,31)^.

**Figure 13:**
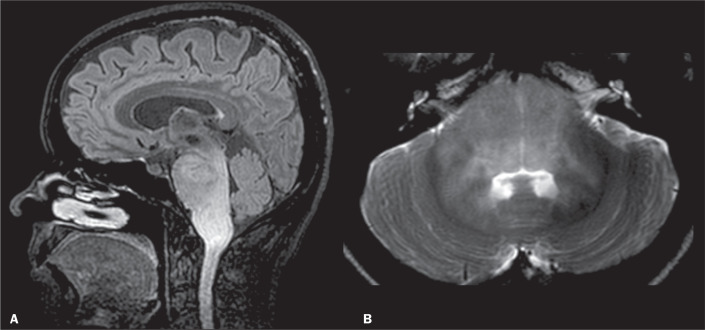
Diffuse midline H3K27M-mutated glioma. Sagittal FLAIR sequence (A) and axial T2WI (B), showing an expansile hyperintense lesion in the pons, and flattening of the fourth ventricle floor involving the medulla oblongata and the MCPs.

Neuroimaging commonly reveals lesions not only in the pons but also in the brainstem, thalamus and spinal cord. These lesions are usually hypointense on T1WI, hyperintense on T2WI, and can present with hyperintensity in the MCP (Figure 13). There can be minimal or no gadolinium enhancement. Tumour size, infiltrative appearance on FLAIR sequences, the mass effect, contrast enhancement characteristics, the presence of necrosis, and the pattern of disease recurrence are similar between wild-type and histone H3K27M-mutated gliomas^(32)^.

## CONCLUSION

A proper imaging investigation is essential for the diagnosis of several neurological diseases, including those described here, which cause alterations specifically in the MCP. Knowledge of the imaging patterns associated with these diseases can help the practicing radiologist make an informed analysis that, in turn, will lead to a more accurate diagnosis by the neurologist and, potentially, better treatment. As such, an adequate understanding of the imaging patterns associated with MCP lesions is essential knowledge for radiologists.
